# Prevalence of Thyroid Autoimmune Antibodies in Women Seeking Fertility Care in Damascus, Syria

**DOI:** 10.7759/cureus.5315

**Published:** 2019-08-03

**Authors:** Mohammad Aljarad, Nawras Alhalabi, Ahed Hamad, Nazht Nmr, Fatima Abbas, Adnan Alkhatib, Marwan Alhalabi, Hisham Al-Hammami, Nazir Ibrahim

**Affiliations:** 1 Miscellaneous, Faculty of Medicine, Syrian Private University, Damascus, SYR; 2 Internal Medicine, Faculty of Medicine, Syrian Private University, Damascus, SYR; 3 Miscellaneous, Faculty of Medicine, Damascus University, Damascus, SYR; 4 Internal Medicine, Faculty of Medicine, Damascus University, Damascus, SYR; 5 Genetics, Clinical Lab Unit, Alkhatib Lab, Damascus, SYR; 6 Obstetrics and Gynecology, Faculty of Medicine, Damascus University, Damascus, SYR; 7 Obstetrics and Gynecology, Faculty of Medicine, Syrian Private University, Damascus, SYR

**Keywords:** thyroid autoimmunity, anti thyroid peroxidase antibodies (anti-tpo), anti thyroglobulin antibodies (anti-tg), anti thyroglobulin antibodies (anti-tg), thyroid stimulating hormone (tsh), syria, syria

## Abstract

Introduction

Thyroid autoimmune (TAI) disease with a prevalence varying between 5 and 15%, represents the most common endocrine disorder in women with reproductive age. Not only is TAI disease five to 10 folds more common in women than men but also TAI diseases is often undiagnosed because it may be present without overt thyroid dysfunction for several years. Studies found an increased prevalence of TAI in women referred to fertility clinics compared with normal population. In this analysis we aimed to study the prevalence of TAI among women seeking fertility care in Damascus, Syria in order to understand its clinical and public health importance in population.

Methods

This study is a retrospective cross-sectional study on women patients seeking fertility care at Orient Hospital, Damascus city, Syria from April 2011 to March 2018. A total of 2526 women, with available biochemical data of anti-thyroid antibodies (anti-TPO) and anti-thyroglobulin antibodies (anti-TG) were included in our study. Thyroid stimulating hormone (TSH) titers data were also included in the statistical analysis.

Results

TAI was found positive in 559 patients (22.1%) of our studies population. TAI was more prevalent in patients with abnormal TSH levels.

Conclusion

Thyroid autoimmunity prevalence in women seeking fertility care in Damascus, Syria was 22.1% which is significantly higher than normal population. Further studies are needed to assess the relation of these antibodies in thyroid, gynecological and other factors for the Syrian population.

## Introduction

Chronic morbidity and impairment are caused by autoimmune diseases, and the thyroid gland is the most commonly affected organ by these diseases [1]. Thyroid autoimmunity (TAI) occurs mostly in females more than males and is generally consisted of two major types of diseases, Graves’ disease, and Hashimoto’s thyroiditis with diverse pathogenic mechanisms [2]. The immunological mechanisms concerned in these diseases are correlated, while the phenotypes may vary because of the difference between the specific types of immunological response that occurs [3].

Hyperthyroidism is caused by Graves' disease which has an approximated prevalence of 80/100,000/year in women and 8/100,000/year in men in Western countries, while Hashimoto's disease is considered the main cause of hypothyroidism in the West [2]. Intolerance of self-antigens of the thyroid is the main cause of TAI. It seems to happen in several ways including infection, genetic predisposition and abnormal iodine diet [4]. Either form of TAI is associated with and diagnosed by the presence of anti-thyroid autoantibodies (autoantibodies targeted against one or more component of the thyroid), such as serum anti-thyroid peroxidase (TPO) and anti-thyroglobulin (Tg) antibodies even without clinical autoimmune disease [5, 6].

Anti-thyroid peroxidase (anti-TPO) antibodies are distinct for the autoantigen TPO, a 105-kDa glycoprotein that is responsible for catalyzation of iodine oxidation and thyroglobulin tyrosyl iodination reactions in the thyroid [7].

Anti-TPO antibodies present in almost 90% of Hashimoto's thyroiditis which makes them the most common anti-thyroid autoantibodies, in addition around 75% of Graves' disease and 10-20% of nodular goiter or thyroid carcinoma have positive anti-TPO antibodies. Also, 10-15% of normal individuals can have high-level anti-TPO antibody titers [8].

Thyroglobulin antibodies are specific for thyroglobulin, a 660-kDa matrix protein that contributes to the production of thyroid hormone. Seventy percent of Hashimoto's thyroiditis, 60% of idiopathic hypothyroidism, 30% of Graves' disease, a small proportion of thyroid carcinoma and 3% of normal individuals have positive anti-TG antibodies [5].

Recently the prevalence of other autoimmune endocrine disorders, particularly type 1 diabetes mellitus, has considerably increased [9]. While the underlying cause of this increasing prevalence is still poorly understood, this motivates us to investigate whether TAI has the same tendency [6]. It is crucial to be aware of the prevalence of a disease in order to identify the trends in relation to patient characteristics such as sex and geographical regions and to figure out any changes in incidence meeting with any new environmental factors [2].

TAI has also been accused of increased unfavorable pregnancy outcomes, including implantation failure, recurrent pregnancy loss, placental abruption, preterm birth and perinatal mortality [10]. 5-20% of women in reproduction age are shown to be affected by TAI, which is considered positive by the presence of anti-TPO and/or anti-TG antibodies [11]. Not only TAI diseases represent the most common endocrine disorders in women with reproductive age, but also they are often undiagnosed because it may be present without overt thyroid dysfunction for several years [12].

TAI was also recently accused of negative outcomes on reproductive biology, including spermatogenesis, folliculogenesis, fertilization rates (FRs), embryo quality and pregnancy rates [13]. Subsequently, TAI is found more in women in childbearing age who attend fertility clinics compared to the general population [14]. Consequently, thyroid diseases affecting women constitute a great burden of fertility and natal care. As a result, thyroid function is commonly assessed during the reproductive period [15].

So far, in this analysis we aimed to study the prevalence of TAI among women seeking fertility care in Damascus, Syria in order to understand its clinical and public health importance in population. To the best of our knowledge, this study is the first to investigate thyroid autoimmunity burden in Syria.

## Materials and methods

Design

This study is a retrospective cross-sectional study on women patients seeking fertility care at Orient Hospital, Damascus city, Syria from April 2011 to March 2018. Participants were referred by their physician for the screening of their thyroid issues relating to fertility and/or gynecological issues.

Study population

A total of 2526 women, with available biochemical data of anti-thyroid antibodies (anti-TPO) and anti-thyroglobulin antibodies (anti-TG), were included in our study. Thyroid stimulating hormone (TSH) titers data was also included in the statistical analysis.

Immune assays

All tests were done in the same lab (Alkhatib Lab, Damascus, Syria). All tests were done using the same immune assay. These were primarily measured on the instrument Cobas 6000 (Roche Diagnostics). The instrument is a closed system, random access auto-analyzer. Performance characteristics of measurement previously explained [16]. The study was spaced out into a whole calendar year deliberately to even out the variations brought in by changes in lots of reagents, calibrators and in instrument calibrations. In particular, two lots of calibrators were used in each instrument, four lots of reagent for Cobas were used during this period. Method calibrations were done on instrument roughly once every month for a change of lots. Two levels of 3rd party commercial control materials were run on every working day.

Electrochemiluminescence (ECL) is Roche’s technology for immunoassay detection. Based on this technology and combined with well-designed, specific and sensitive immunoassays, Elecsys delivers reliable results. The development of ECL immunoassays is based on the use of a ruthenium-complex and tripropylamine (TPA). The chemiluminescence reaction for the detection of the reaction complex is initiated by applying a voltage to the sample solution resulting in a precisely controlled reaction. ECL technology can accommodate many immunoassay principles while providing superior performance.

Lab tests cutoff values were considered as follows: TPO antibodies ≥ 35 IU/ml, TG antibodies ≥ 40 IU/ml, TSH was defined as ‘abnormally low’ (<0.45), ‘low normal’ (0.45-2.5), ‘high normal’ (2.5-4.5) and ‘abnormally high’ (>4.5) uIU/ml.

Statistical analysis

Thyroid autoimmunity status was considered positive in the presence of anti-TPO and/or anti-TG higher than the upper limit of the reference range. Subgroup analysis for TSH levels between 0.45 and 2.5 was defined as low normal, and between 2.5 and 4.5 was defined as high normal [17]. Statistical analysis was done using the Statistical Program for Social Sciences (Version 25; IBM Corp., Armonk, NY, USA). Descriptive data are summarized as frequencies, Mann-Whitney U test, Kruskal-Wallis H test were used as appropriate. The value of p < 0.05 was considered statistically signiﬁcant.

Ethical consideration

This study was approved by Syrian Private University Research Committee with the approval of the Orient Hospital Board of Directors.

## Results

A total of 2526 women who referred to our fertility center were studied for the presence of TAI. TAI was considered positive in 559 patients (22.1%) of our studies population (Table 1).

The mean age for TAI positive patients was 30.02 ± 0.65 years (mean ± SD, range: 22-37) while it was 30.10 ± 0.10 (range: 18-43) in TAI negative group (p-value = 0.818) (Table 1).

**Table 1:** Patients characteristics. TAI: Thyroid autoimmune

	TAI Positive	TAI Negative	Total	p-value
	n (%)	n (%)	n (%)	
Patients' No.	559 (22.1)	1967 (77.9)	2526 (100.0)	
Age (years)	30.02 ± 0.65	30.10 ± 0.10	30.09 ± 1.05	0.818

The prevalence of TAI positivity was significantly higher in abnormally low TSH patients (41.18%, n = 7) and in abnormally high TSH patients (53.33%, n = 24) than patients with TSH levels within normal ranges (p-value < 0.001) (Table 2).

In addition, subgroup analysis between low normal (0.45-2.5 uIU/ml) and high normal (2.5-4.5 uIU/ml) TSH levels groups revealed that TAI positivity was higher in high normal TSH levels group (36.61%, n = 41) than in low normal TSH levels group (16.12%, n = 44) (p-value < 0.001) (Table 2).

**Table 2:** Thyroid autoimmunity and TSH levels (uIU/ml). *p-value calculated only between low normal and high normal TSH levels groups. TSH: Thyroid stimulating hormone; TAI: Thyroid autoimmune.

	TAI Positive	%	TAI Negative	%	Total	% of total	p-value
Abnormally Low	7	41.2%	10	58.8%	17	3.8%	<0.001
Low Normal	44	16.1%	229	83.9%	273	61.1%	<0.001*
High Normal	41	36.6%	71	63.4%	112	25.1%	
Abnormally High	24	53.3%	21	46.7%	45	10.1%	

When doing subgroup analysis for TSH levels, there was a significant difference between the mean age for abnormally low TSH levels group (30.00 ± 0.00 years), for low normal TSH levels group (30.08 ± 1.17 years), for high normal TSH levels group (30.17 ± 1.26 years) and for abnormally high TSH levels group (30.28 ± 1.79 years) (p-value = 0.009). While, when comparing the mean age of low normal TSH levels group with the mean age of high normal TSH levels group, no difference was found (p-value = 0.378) (Table 3).

**Table 3:** TSH levels (uIU/ml) and age. *p-value calculated only between low normal and high normal TSH levels groups. TSH: Thyroid stimulating hormone

	n	Age (years)	±	SD	p-value
Abnormally Low	17	30.00	±	0.00	0.009
Low Normal	273	30.08	±	1.17	0.378*
High Normal	112	30.17	±	1.26	
Abnormally High	45	30.28	±	1.79	

The difference in the average age of the patients admitted in different years is statistically significant (p-value < 0.001) (Table 4). Among the years of lab tests, no differences were noticed between TSH level groups (p-value = 0.084) (Table 5).

**Table 4:** Age according to years of lab tests.

	n	Age (years)	±	SD	p-value
2011	433	30.04	±	0.15	<0.001
2012	289	30.03	±	0.11	
2013	165	30.03	±	0.13	
2014	427	30.01	±	0.07	
2015	404	30.00	±	0.05	
2016	330	30.01	±	0.08	
2017	363	30.33	±	2.24	
2018	115	30.55	±	2.80	

**Table 5:** TSH levels (uIU/ml) according to years of lab tests. *p-value calculated only between low normal and high normal TSH levels groups. TSH: Thyroid stimulating hormone

	Abnormally Low	%		Low Normal		%	High Normal		%		Abnormally High	%		Total		(% of all)
2011	1	1.4%		44		59.5%	17		23.0%		12	16.2%		74		14.8%
2012	4	8.2%		33		67.3%	6		12.2%		6	12.2%		49		9.8%
2013	1	3.2%		21		67.7%	8		25.8%		1	3.2%		31		6.2%
2014	3	4.5%		46		69.7%	13		19.7%		4	6.1%		66		13.2%
2015	0	0.0%		36		66.7%	13		24.1%		5	9.3%		54		10.8%
2016	5	7.0%		43		60.6%	19		26.8%		4	5.6%		71		14.2%
2017	2	2.4%		42		50.6%	30		36.1%		9	10.8%		83		16.6%
2018	1	5.3%		8		42.1%	6		31.6%		4	21.1%		19		3.8%
p-value	0.084				0.099*											

**Figure 1:**
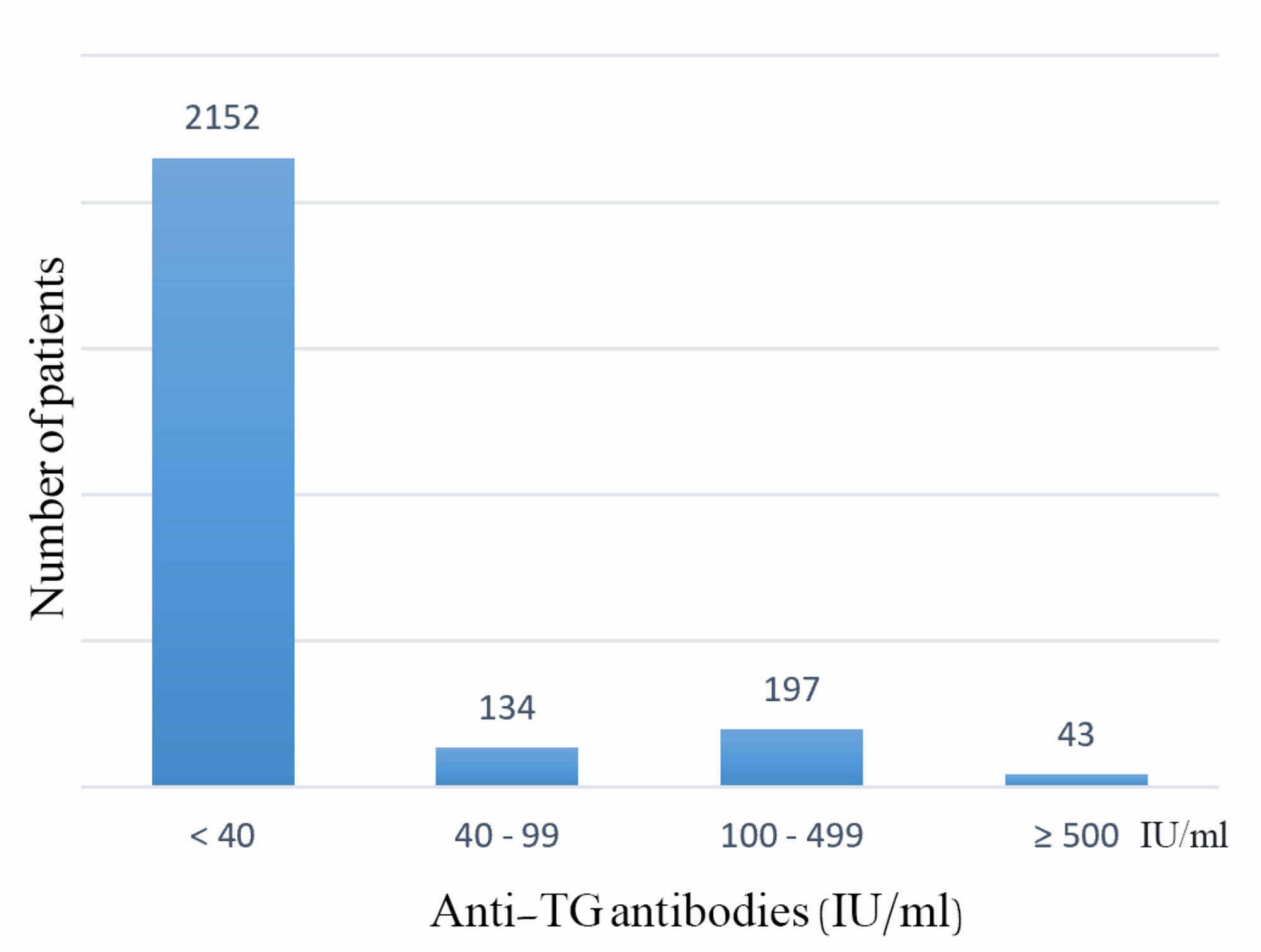
Anti-TG antibodies titers. Anti-TG: Anti-thyroglobulin

**Figure 2:**
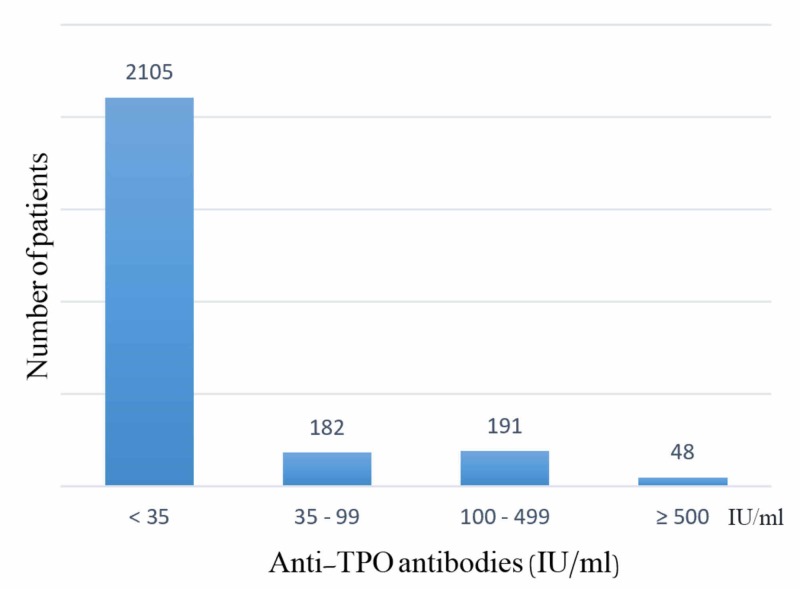
Anti-TPO antibodies titers. Anti-TPO: Anti-thyroid peroxidase

Finally, when detecting the prevalence of TAI positivity according to the years of lab tests we established that it was 28.18% (n = 122) in 2011, 31.83% (n = 92) in 2012 but 15.10% (n = 61) in 2015 (p-value < 0.001) (Table 6). Anti-TPO and anti-TG levels were classified according to antibody titers as shown in Figure 1 and Figure 2.

**Table 6:** Thyroid autoimmunity according to years of lab tests. TAI: Thyroid autoimmune

	TAI Positive	%	TAI Negative	%	Total
2011	122	28.2%	311	71.8%	433
2012	92	31.8%	197	68.2%	289
2013	43	26.1%	122	73.9%	165
2014	80	18.7%	347	81.3%	427
2015	61	15.1%	343	84.9%	404
2016	57	17.3%	273	82.7%	330
2017	83	22.9%	280	77.1%	363
2018	21	18.3%	94	81.7%	115
p-value	<0.001				

## Discussion

To the best of our knowledge, this is the first study in Syria to investigate the prevalence of thyroid autoimmunity in women with fertility issues.

Studies found an increased prevalence of TAI in women referred to fertility clinics compared with the normal population [18]. Our investigation resulted in a proportion of 22.1% of women to have abnormal thyroid antibodies in our center. In McGrogan et al. study, researchers found that TAI prevalence in general population was between 8 and 14% which is much less than women seeking fertility care including our percentage [2, 18]. In addition, a study by Boufas et al. showed that anti-TPO titers were much higher in women with no previous pregnancies compared with women with one or those with at least two pregnancies [19]. These women form the couples seeking fertility care. Moreover, Korevaar et al. showed that TAI is even involved in low ovarian reserve in this population [20]. Increased number of women having positive thyroid dysfunction among those seeking fertility care may indicate an association between thyroid autoimmunity and poor obstetric outcomes as shown by Glinoer [21].

Thyroid hormones interfere with numerous aspects of reproduction. Normal ovarian function and pregnancy outcomes are adversely affected by hypothyroidism and hyperthyroidism. Actually, since the early 1990s, many studies about the influence of thyroid autoantibodies on recurrent miscarriages and infertility in euthyroid women have been published [21, 22]. They finally concluded that recurrent abortions and failure to conceive are associated with increased positivity of thyroid autoantibodies [22].

On the other hand, some studies investigated different treatment modalities for infertility in women with positive TAI [23]. Thus, although the available evidence is still not definitive in determining the relation between TAI and infertility, investigation of thyroid dysfunction in infertile women might be of high benefit once required in the assessment of infertility risk factors and causes.

From another perspective, comparing mean age between positive and negative TAI showed no significant difference between the two groups (p = 0.818), this is consistent with the study done by Poppe et al. [24]. A study by Kontiainen et al. found an increased prevalence of anti-TPO with age [25]. Another study by Kutteh et al. demonstrated elevated antithyroid antibodies titers with age until the age range of 31-35 years, after which titers found to be decreased [26]. In Anderson et al. study as in our study, age was ruled out as a modifier despite the fact that some studies have shown it to have an impact on TAI presence [27].

TSH levels in our study showed a significantly higher prevalence of TAI in abnormally low and abnormally high TSH levels. Similarly, TSH abnormally high were frequently more reported in the positive group in comparison to negative TAI women than abnormal low TSH. This finding was previously established [25].

TSH level in correlation with TAI in the pathophysiology of infertility is still controversial. Small case-control prospective studies and a recent systematic review, which included retrospective studies, resulted in no association of TAI and assisted reproductive techniques outcomes, while others found the contrary [28-30].

One limitation that prevented to provide a definitive comparison of our results with other studies is using different immune assays, different lab methods in TAI measurements as suggested in previous studies [23, 29]. We in our study, have overcome this obstacle by measuring all lab results at the same laboratory, using the same methods, and with standardizing the immune assay. In addition, we could not include overt and subclinical hypothyroidism and hyperthyroidism patients as our data did not include T3, free T3, T4 or free T4 laboratory tests. Further studies are also needed to include other immunological antibodies such as anti-thyroid stimulating hormone receptors and other immunological antibodies.

## Conclusions

Thyroid autoimmunity prevalence in women seeking fertility care in Damascus, Syria was 22.1% which is significantly higher than the normal population. Further studies are needed to assess the relation of these antibodies in the thyroid, gynecological and other factors for the Syrian population.
